# Revisiting the Role of VraTSR in *Staphylococcus aureus* Response to Cell Wall-Targeting Antibiotics

**DOI:** 10.1128/jb.00162-22

**Published:** 2022-07-13

**Authors:** Pedro B. Fernandes, Patricia Reed, João M. Monteiro, Mariana G. Pinho

**Affiliations:** a Instituto de Tecnologia Química e Biológica António Xavier, Universidade Nova de Lisboa, Oeiras, Portugal; University of Illinois at Chicago

**Keywords:** *Staphylococcus aureus*, antibiotic resistance, peptidoglycan synthesis, two-component system

## Abstract

Exposure of Staphylococcus aureus to cell wall inhibitors leads to the activation of the VraTSR three-component sensory regulatory system. This system is composed of VraS, a membrane histidine kinase; VraR, its cognate response regulator, and VraT, a protein required for the full activity of VraTSR. The exact function of VraT remains mostly uncharacterized, although it has been proposed to detect the unknown stimulus sensed by the VraTSR system. Here, we elucidate the topology of VraT, showing that its C-terminal domain is extracellular. We also demonstrate that the signal sensed by VraTSR is not an intermediate in the peptidoglycan synthesis pathway, as previously suggested. Instead, the specific inhibition of the penicillin-binding protein (PBP)2 leads to strong activation of the system.

**IMPORTANCE** The Gram-positive bacterial pathogen Staphylococcus aureus is currently the second most frequent cause of global deaths associated with antibiotic resistance. Its response to cell wall-targeting antibiotics requires the VraTSR three-component system, which senses cell wall damage. Here, we show that the signal sensed by VraTSR is not an intermediate in the peptidoglycan synthesis pathway, as previously suggested. Instead, the specific inhibition of the penicillin-binding protein (PBP)2, the major peptidoglycan synthase in S. aureus, leads to strong activation of the system. Identifying the exact cell wall damage signal is key to fully understanding the response of S. aureus to cell wall-targeting antibiotics.

## INTRODUCTION

Staphylococcus aureus is a Gram-positive bacterial pathogen responsible for nosocomial and community-acquired infections worldwide. Methicillin-resistant S. aureus (MRSA) strains in particular are currently the second most frequent cause of global deaths associated with antibiotic resistance (1). S. aureus can cause a variety of infections in the human body, ranging from mild skin infections to life-threatening diseases (2). The versatility of this pathogen is partially due to its capacity to cope with changing environmental conditions, with adaptative responses coordinated by its 16 two-component systems (TCS) (3).

Bacterial TCS sense various environmental cues, including different chemical and physical stimuli such as ions, gases, antibiotics, osmotic pressure, and metabolites (4, 5). This allows bacterial cells to mount the appropriate response via changes in metabolism, cell division, antibiotic resistance, and pathogenicity, among other functions (6).

Among the several TCS present in S. aureus, the vancomycin resistance-associated regulatory system, also known as VraTSR, coordinates the bacterial response to cell wall (CW) synthesis disruption (7). VraTSR was initially identified due to its role in vancomycin resistance. However, it is also involved in the responses to several other compounds which target CW synthesis (8–10). Accordingly, disrupting this system increases the susceptibility of S. aureus toward several CW-targeting compounds (8).

VraTSR is encoded by the *vraUTSR* operon (7). VraS is a membrane histidine kinase belonging to the intramembrane-sensing histidine kinase (IMHK) family, since it is predicted to lack an extracellular sensing domain, which is characteristic of most histidine kinases (7, 11, 12). Upon detecting a still unknown stimulus, presumably resulting from S. aureus exposure to CW-targeting compounds, VraS undergoes autophosphorylation at a conserved histidine residue, likely Histidine 156 (11, 13). VraR is the cognate response regulator that is phosphorylated by VraS (11). Phosphorylated VraR dimerizes and binds to its own *vraUTSR* promoter, as well as to the promoters of several other genes, activating or repressing their transcription (11, 14). These genes, which constitute the VraR regulon, also known as the cell wall stress stimulon (CWSS) (15), belong to different functional categories, including lipid and carbohydrate metabolism, DNA replication and repair, and importantly, cell envelope biogenesis. The latter encompasses genes involved in peptidoglycan biosynthesis, such as *pbpB*, encoding the major S. aureus peptidoglycan synthase penicillin-binding protein 2 (PBP2); *mgt*, encoding a monofunctional glycosyltransferase; and *murZ*, which encodes a UDP-*N*-acetylglucosamine enolpyruvyl transferase (8, 9).

The role of VraT is less clear. Boyle-Vavra et al. have proposed that VraT (previously named YvqF) is the protein which detects the unknown stimulus sensed by the VraTSR system, promoting autophosphorylation of VraS, through a still-elusive mechanism (7). Accordingly, deletion of *vraT* affects the resistance of S. aureus to several CW-targeting antibiotics, similarly to what was observed for *vraS* and *vraR* deletion mutants (7, 16). Deletion of *vraT* also impairs VraTSR-dependent activation of the CWSS, showing its relevant role in this process (7, 16). VraT is predicted to be a membrane protein, described as interacting with VraS, but not VraR (7, 16). Its structure and topology are not known, and therefore the cellular localization of the C-terminal region, speculated to be the sensor domain, is yet to be determined (7, 16).

In this work, we aimed to unveil the identity of the stimulus responsible for VraTSR activation, specifically to determine whether this stimulus was an intermediate in the peptidoglycan synthesis pathway that would accumulate, or be depleted, in the presence of various CW-targeting antibiotics.

## RESULTS

### VraT is a membrane-localized protein with an extracellular C-terminal domain.

**FIG 1:**
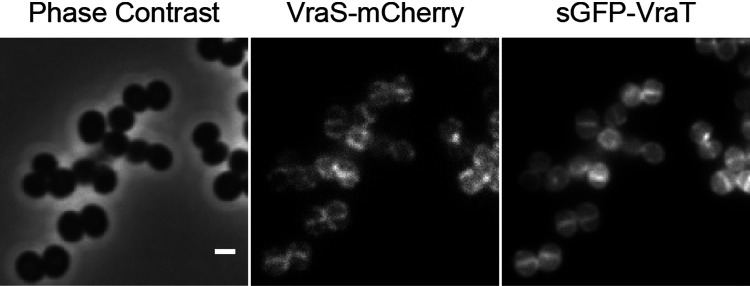
VraT and VraS localize at the cell membrane. Phase contrast (left) and epifluorescence microscopy images of S. aureus COL expressing a mCherry-tagged derivative of VraS (middle) and a sGFP-tagged derivative of VraT (right). The VraTSR system was induced by incubating the cells for 60 min with 0.5 μg · mL^−1^ of CDFI, an inhibitor of the lipid II flippase MurJ. Scale bar 1 μm.

The role of VraT has remained elusive, but it has been suggested to be involved in stimulus perception of the VraTSR regulatory system (7). We confirmed that both VraT and VraS localize to the membrane by using fluorescent fusions to both proteins (Fig. 1). This result indicates that, as expected, CW damage is perceived either at the cell surface or at the cell membrane.

**FIG 2:**
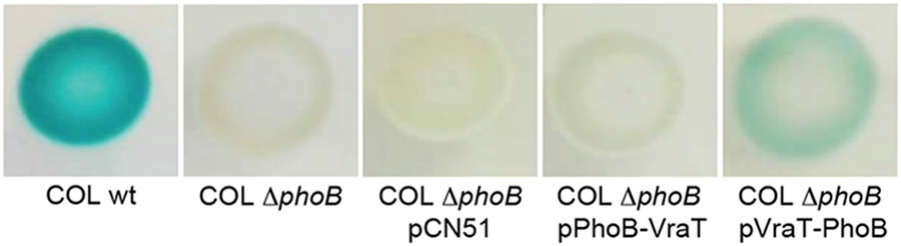
C-terminal domain of VraT is extracellular. VraT topology was assessed by fusing the *phoB* gene to *vraT* at either end. The *phoB* fusions to *vraT* were expressed from the pCN51 plasmid, under the control of a cadmium-inducible promoter, in the background of a *phoB* deletion mutant (COLΔ*phoB*). An empty pCN51 plasmid was also introduced into COLΔ*phoB* (COLΔ*phoB* + pCN51) and used as a negative control. The wild-type strain COL was used as positive control. The strains were grown on plates containing cadmium chloride and chromogenic BCIP (5-bromo-4-chloro-3-indolyl phosphate), a substrate which, when processed by the alkaline phosphatase PhoB, an enzyme active only in the extracytoplasmic environment, gives a blue coloration to the colonies (41). Only COL and COLΔ*phoB* carrying the *vraT*-*phoB* fusion (COLΔ*phoB* + pVraT-PhoB) showed alkaline phosphatase activity, indicating that the C-terminal domain but not the N-terminal domain of VraT faces the extracytoplasmic environment.

Given that VraS, initially thought to be the sensor of the VraSR two-component system, does not display a clear extracellular sensing domain, characteristic of most histidine kinases, Boyle-Vavra et al. suggested that VraT was responsible for perception of CW damage on the outer surface of the cell (7, 12). However, the topology of VraT remained unclear, with different authors suggesting that its C-terminal domain lies intracellularly or extracellularly (7, 16). To examine the topology of VraT, we fused the *phoB* gene, encoding staphylococcal alkaline phosphatase, lacking its native export signal peptide, to either end of *vraT* and evaluated the enzymatic activity, as previously described (17). The alkaline phosphatase PhoB is active only in the extracytoplasmic environment, where it can process the chromogenic alkaline phosphatase substrate BCIP (5-bromo-4-chloro-3-indolyl phosphate), generating a blue color. As shown in Fig. 2, only the PhoB C-terminal fusion to VraT showed some PhoB activity, indicating that the C-terminal domain of VraT is extracellular, while the N-terminal region is intracellular. This topology is compatible with a sensor function for the C-terminal domain of VraT.

### VraTSR does not sense an intermediate in peptidoglycan synthesis.

**FIG 3:**
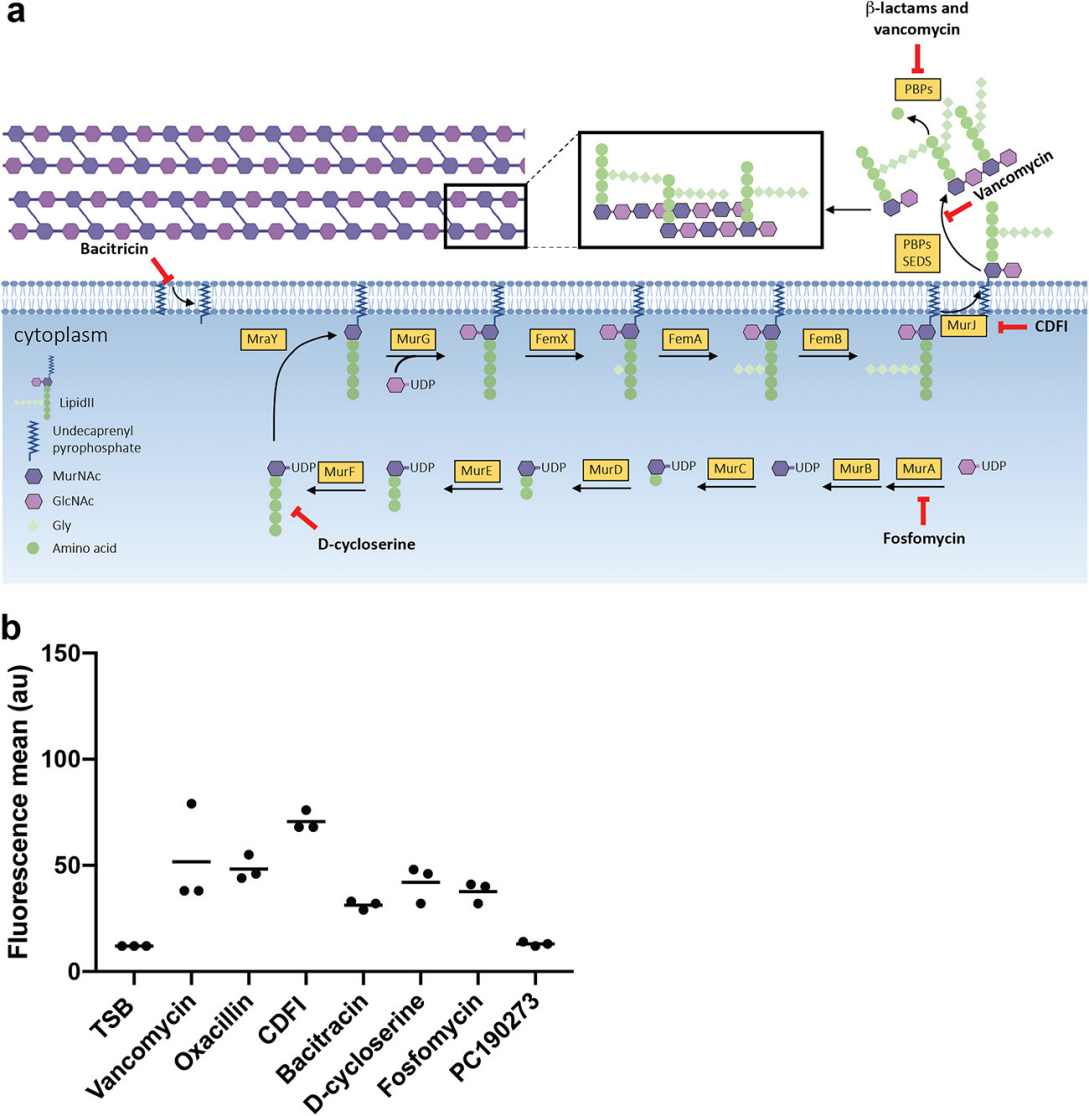
VraTSR responds to various cell wall (CW)-targeting antibiotics (a) Schematic representation of peptidoglycan synthesis pathway, indicating the targets of cell wall active antibiotics. Fosfomycin inhibits the enzyme MurA (UDP-*N*-acetylglucosamine-3-enolpyruvyl transferase) that performs the addition of phosphoenolpyruvate to UDP-*N*-acetylglucosamine (GlcNAc), generating UDP-*N*-acetyl-muramic acid (UDP-MurNAc) (42). d-Cycloserine inhibits the addition of d-alanine to the peptidoglycan precursor by inhibiting d-alanine:d-alanine ligase A and alanine racemase (43). CDFI inhibits MurJ (44), the lipid II flippase. Bacitracin blocks dephosphorylation and recycling of the lipid carrier undecaprenyl pyrophosphate, preventing the membrane steps of peptidoglycan synthesis (45). Glycopeptide antibiotics, such as vancomycin, bind to the d-Ala-d-Ala motif of lipid II, blocking the access of enzymes performing transglycosylase and/or transpeptidase activity (46). β-Lactam antibiotics, such as oxacillin, mimic the d-Ala-d-Ala end of lipid II stem peptide, forming an acyl-enzyme complex with PBPs, inhibiting their activity (47). (b) COL Pvra-sGFP cells, which express green fluorescent protein (GFP) under the control of the *vraTSR* promoter, were incubated with 1× MIC of different CW-targeting antibiotics for 60 min prior to analysis by fluorescence-activated cell sorting (FACS). All tested CW-targeting antibiotics activated the VraTSR system independently of the blocked step of the PG synthesis pathway. Incubation with the FtsZ inhibitor PC190273, an antibiotic that does not target CW synthesis, was included as a negative control. TSB indicates growth in the absence of antibiotics. *N* = 5,000 cells for each replicate; experiments were done in triplicate. Each point represents the median of one FACS experiment.

CW synthesis is a multi-enzymatic, sequential process that leads to the formation of the lipid II molecule (Fig. 3a) (18). Lipid II is subsequently flipped to the extracellular space and inserted in the peptidoglycan (PG) mesh (18). Thus, the accumulation or depletion of an intermediate molecule in this enzymatic pathway, particularly lipid II, would be a good candidate for the signal sensed by VraTSR. We tested the effects of various antibiotics blocking the initial or final steps of the PG synthesis pathway (Fig. 3a) and determined that in all cases, the VraTSR response was triggered (Fig. 3b and Fig. S1 in the supplemental material), confirming previous work (10). This was done by incubating COL Pvra-sGFP cells, where GFP expression is driven by the *vraTSR* promoter, with 1× the MICs of different CW-targeting antibiotics for 60 min and subsequently analyzing the fluorescence of the cells by fluorescence-activated cell sorting (FACS). Antibiotics blocking the early stages of CW synthesis, like fosfomycin, are expected to inhibit the formation of all subsequent lipid precursors, including lipid II, while antibiotics blocking the late steps of this process, like vancomycin, were previously shown to accumulate lipid II (19, 20). Considering that these antibiotics produce opposite effects on PG precursor levels, but all result in the activation of the VraTSR response, it seems unlikely that VraTSR is sensing the accumulation or depletion of lipid-linked PG precursors.

### Inhibition of transglycosylase activity induces VraTSR.

**FIG 4:**
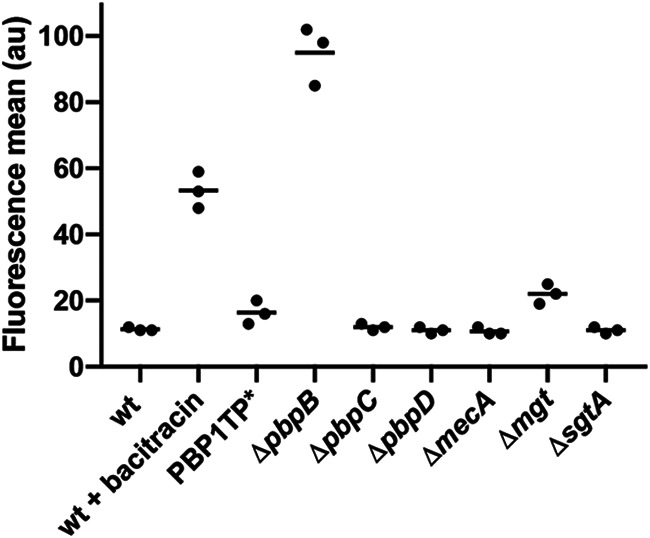
Depletion of PBP2, but not of the remaining CW synthesis enzymes, leads to VraTSR activation. S. aureus mutants lacking different CW synthesis enzymes, PBP2 (Δ*pbpB*), PBP3 (Δ*pbpC*), PBP4 (Δ*pbpD*), PBP2A (Δ*mecA*), MGT (Δ*mgt*), or SgtA (Δ*sgtA*), or encoding a transpeptidase mutant of the essential PBP1 (PBP1TP*) and expressing GFP under the control of the *vraTSR* promoter, were analyzed by FACS. Only the cells lacking PBP2 (Δ*pbpB*) showed strong VraTSR activation, while the Δ*mgt* mutant showed low activation levels. Control experiments, where COL Pvra-sGFP cells were grown in the absence (wt) or presence of bacitracin (wt + bacitracin), which induces the VraTSR response, were performed as negative and positive controls, respectively. *N* = 5,000 cells for each replicate; experiments were done in triplicate. Each point represents the median of one FACS experiment.

Given that we were unable to identify one specific CW synthesis precursor as the signal that triggers the VraTSR system, we decided to investigate whether the system was triggered by lack of activity of a particular enzyme involved in CW synthesis. We started by focusing on the late stages of CW synthesis performed by PBPs (1 to 4), the exogenous PBP (PBP2A), and the monofunctional glycosyltransferases MGT and SgtA (21). Two of these enzymes, the bifunctional transpeptidase-transglycosylase PBP2 and the monofunctional transglycosylase MGT, have their genetic expression upregulated by the response regulator VraR as part of the CWSS (8, 9). Deletion of the gene encoding PBP2, but not of those encoding other PBPs, led to the activation of VraTSR (Fig. 4). PBP2 is the only bifunctional PBP in S. aureus, while the remaining PBPs display monofunctional transpeptidase activity (21). These results are in line with previous work showing that decreasing levels of *pbpB* (encoding PBP2) transcription lead to increasing levels of *vraTSR* transcripts (22).

**FIG 5:**
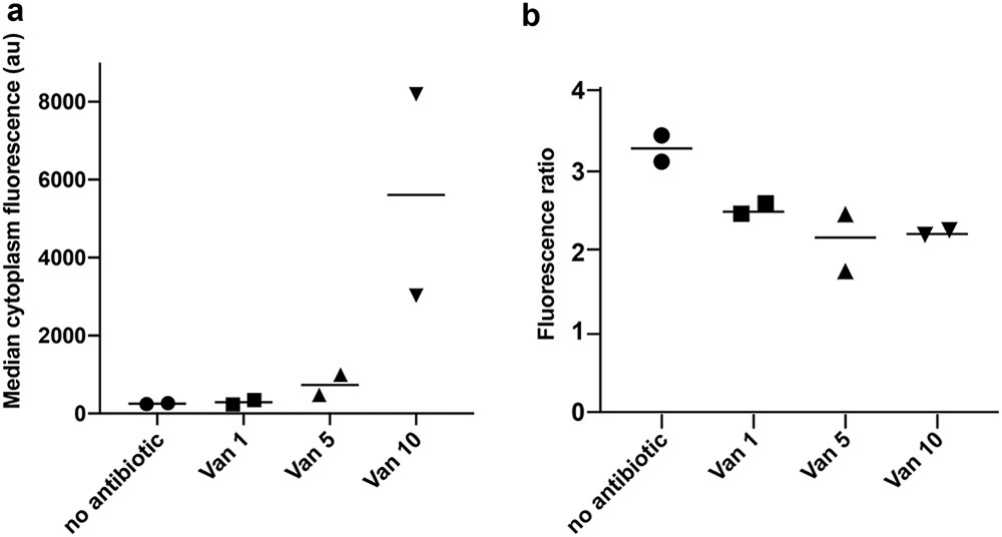
PBP2 delocalization does not correlate with VraTSR activation. Strains COL Pvra-sGFP and BCBPM073 (expressing a GFP fluorescent derivative of PBP2) were both incubated in the presence of increasing vancomycin concentrations (Van 0, 1, 5, and 10 μg · mL^−1^) for 45 min. COL Pvra-sGFP cells were then stained with DNA dye Hoechst 33342 to differentiate the two populations and mixed with BCBPM073 cells just prior to imaging on the same microscope slide. (a) GFP fluorescence of COL Pvra-sGFP cells, showing that VraTSR activation occurs only in the presence of 10 μg · mL^−1^ of vancomycin. *N* > 277 cells for each condition in each replicate. (b) PBP2 septal enrichment was evaluated in BCBPM073 cells by measuring the fluorescence ratio of GFP-PBP2 fluorescence at the septum versus at the cell periphery, both corrected for background fluorescence. PBP2 delocalized from the septum at the lowest vancomycin concentration tested. *N* = between 62 and 90 for each condition in each experiment; experiments performed in duplicate.

To explore the mechanism behind PBP2-dependent VraTSR activation, we evaluated whether the delocalization of PBP2 from the division septum was the signal detected by VraTSR. PBP2 is recruited to the division septum by binding to its substrate, lipid II (23), and the presence of CW synthesis inhibitors causes PBP2 delocalization (Fig. S2 in the supplemental material) in agreement with previous observations (23, 24). To test this hypothesis, we incubated cells expressing a fluorescent derivative of PBP2 (strain BCBPM073) and cells expressing green fluorescent protein (GFP) under the control of the *vraTSR* promoter (COL Pvra-sGFP) with increasing concentrations of vancomycin. Interestingly, we observed that PBP2 delocalized with concentrations as low as 1 μg · mL^−1^ of vancomycin, while VraTSR remained untriggered at that concentration (Fig. 5). In the experimental settings tested, we could only observe VraTSR activation when we incubated the cells with 10 μg · mL^−1^ of vancomycin, a concentration 10-fold higher than that required to delocalize PBP2 from the septum (Fig. 5), indicating that PBP2 delocalization alone is not the trigger for the VraTSR system.

**FIG 6:**
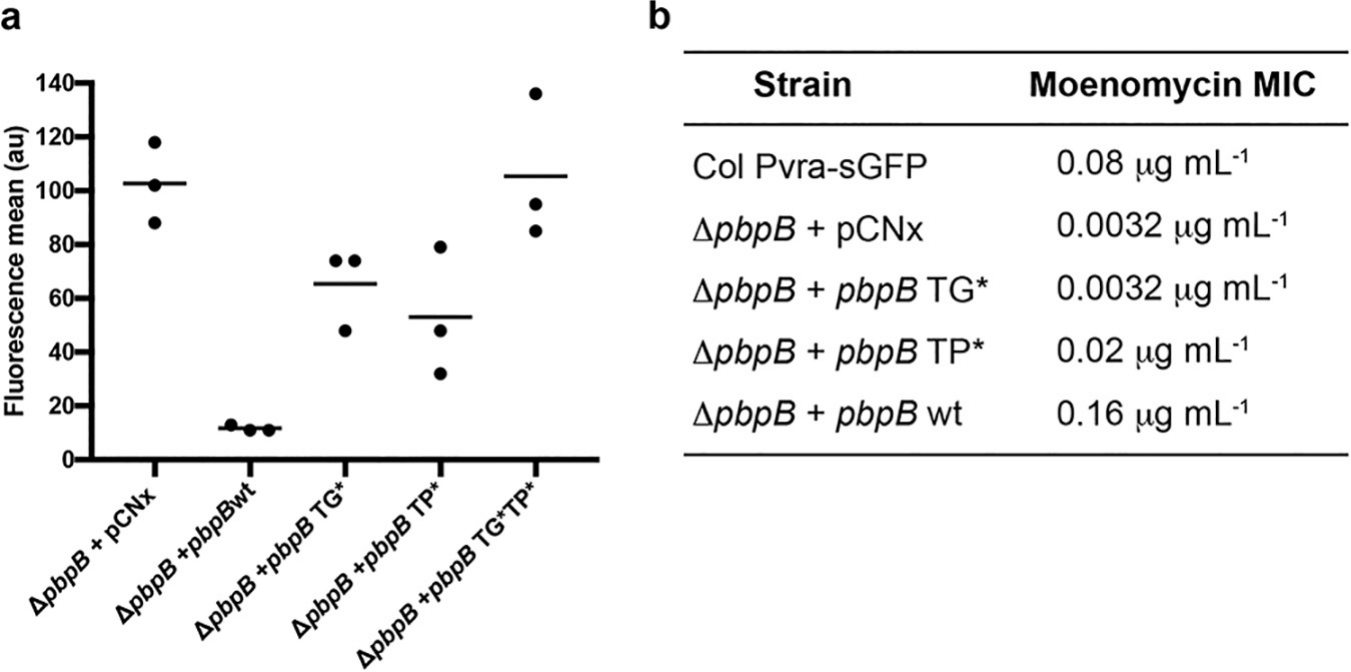
PBP2 transglycosylase and transpeptidase activities are both important for preventing VraTSR activation. (a) Strain COLΔ*pbpB_*Pvra-sGFP was complemented with plasmids encoding either the wild-type allele of *pbpB* (Δ*pbpB* + *pbpB wt*) or mutated alleles encoding a transglycosylase inactive PBP2^E114Q^ (Δ*pbpB* + *pbpB* TG*), a transpeptidase inactive PBP2^S398^ (Δ*pbpB* + *pbpB* TP*), or a double mutant PBP2^E114Q S398^ (Δ*pbpB* + *pbpB* TG*TP*). A control complemented with an empty vector (Δ*pbpB* + pCNX), lacking PBP2, was also included. The VraTSR system was induced in the absence of PBP2 or in the presence of any of the tested PBP2 mutants. *N* = 5,000 cells for each experiment; experiments performed in triplicate. Each point represents the median of one FACS experiment. (b) Moenomycin MICs for strains from panel a, as well as that for the control strain COL Pvra-sGFP.

Because PBP2 delocalization was not the trigger for VraTSR activation, we further explored whether lack of this protein or of one of its two enzymatic activities (transpeptidation and transglycosylation) activated the VraTSR system. For that purpose, the strain lacking PBP2 and containing the *vraTSR* GFP reporter (COLΔ*pbpB_*Pvra-sGFP) was complemented with plasmid-encoded PBP2, as well as plasmid-encoded alleles of PBP2, with point mutations to individually ablate transglycosylation, transpeptidation, or both activities simultaneously. When COLΔ*pbpB_*Pvra-sGFP was complemented with an empty plasmid, VraTSR remained activated (Fig. 6), in agreement with the data shown in Fig. 4 for the *pbpB* mutant. Complementation with the double mutant of *pbpB* had similar results, indicating that it is the lack of PBP2 activity and not of the protein itself that signals CW damage. As expected, complementation with the wild-type *pbpB* allele lowered *vraTSR* expression to levels equivalent to those of the parental strain COL Pvra-sGFP. We then focused on which PBP2 activity was relevant for VraTSR activation. Complementation with the construct coding for the glycosyltransferase inactive PBP2^E114Q^ resulted in induced VraTSR, suggesting that a lack of this activity could be key to activating the system. However, complementation with the transpeptidase-inactive PBP2^S398G^ also induced VraTSR, suggesting that either both activities are required to turn off the system or a mutation in one domain may compromise the activity of the other. In fact, the MIC of moenomycin, an antibiotic that inhibits transglycosylase activity (25), for the mutant complemented with the PBP2 lacking transpeptidase activity, showed increased susceptibility toward this antibiotic compared with COL Pvra-sGFP, although not to the same level as the strain complemented with the PBP2 transglycosylase mutant (Fig. 6b). This indicates that the mutation in the transpeptidase domain of PBP2 may partially impair transglycosylation activity, possibly because the two subunits perform a consecutive enzymatic process in which the transpeptidase accepts the substrate from the transglycosylase, so the transglycosylase activity may be impaired if the transpeptidase is inactive.

Taken together, these results suggest that the inhibition of transglycosylase activity seems to be key in triggering the VraTSR regulatory system. We cannot discard a role for inhibition of transpeptidase activity, but we favor the former hypothesis, since (i) the depletion of the other PBPs (monofunctional transpeptidases) did not activate VraTSR and (ii) COLΔ*mgt*, a strain lacking the monofunctional transglycosylase MGT, showed VraTSR activation, albeit to low levels.

## DISCUSSION

Two-component and three-component systems (TCS) are incredibly versatile, capable of detecting a wide variety of stimuli (26). Among the 16 two-component systems described in S. aureus, VraTSR is responsible for detecting CW damage, although the exact signal that is detected remains unknown (7, 9). Other S. aureus TCS, GraXSR and BraSR, also respond to damage inflicted on the CW (27, 28), with additional partners being required for full activity. In the GraXSR system, which responds to antimicrobial peptides (CAMPs), the sensing mechanism depends on the ABC transporter VraFG, encoded by an operon located directly downstream of *graXRS* (27, 29). CAMPs are sensed by VraFG, and the signal is transduced to GraS through a possible interaction between VraG and GraS (27). BraSR, which responds to bacitracin, upregulates the transcription of two operons encoding ABC transporters, *braDE* and *vraDE*, which act as sensing partner and detoxification module, respectively (28). Interestingly, both GraXSR and BraSR have IMHKs, a feature shared with the VraTSR system (27). The putative sensing partner in the VraTSR regulatory system has been proposed to be the membrane protein VraT, although its structure and function remain undetermined (7). Here, we have shown that VraT localizes at the cell membrane, like the histidine kinase VraS, and that its C-terminal domain is extracellular. Since VraS lacks a typical histidine kinase extracellular sensing domain, the signal detection by VraTSR may be performed by VraT, as previously suggested (7).

Given the importance of VraTSR in the antibiotic resistance of MRSA strains, there is great interest in identifying the signal sensed by this system. It was clear early on that the system does not directly sense antibiotic molecules, given that structurally different molecules which target CW synthesis are capable of inducing VraTSR. The best candidate so far for the CW damage signal has been the accumulation or depletion of the peptidoglycan precursor lipid II (30). This molecule is synthesized on the inner side of the cytoplasmic membrane and then flipped to the outer side of the membrane (18). If accumulation of lipid II triggered the VraTSR system, one would expect its activation in the presence of antibiotics such as vancomycin, which lead to lipid II accumulation (20), but not in the presence of antibiotics such as fosfomycin or d-cycloserine, which lead to its depletion. The reverse would be expected if depletion of lipid II was the CW damage signal. However, all tested CW-targeting antibiotics triggered the VraTSR system, indicating that neither the accumulation nor the depletion of any peptidoglycan synthesis precursor is likely to correspond to this signal.

A common effect of all CW-targeting antibiotics tested is the depletion of the substrate for PBPs. S. aureus has four native PBPs (PBPs 1 to 4), with one extra PBP (PBP2A) from an exogenous origin, with low affinity to beta-lactam antibiotics, which is present in MRSA strains (21). PBP2 is the only bifunctional PBP, with both transglycosylase and transpeptidase activities, while the others are monofunctional TPases (31–36). Two S. aureus monofunctional PBPs, namely, the essential PBP1 and the nonessential PBP3, which only have transpeptidase activity, interact with cognate shape, elongation, division and sporulation (SEDS) transglycosylases, FtsW and RodA, respectively, to synthesize PG (34). Monofunctional glycosyltransferases are also present in S. aureus, namely, MGT and SgtA, both of which are nonessential for cell viability (37, 38). Here, we showed that when we individually depleted various PG synthases, only the absence of PBP2 led to strong VraTSR activation, while lack of MGT lead to low-level activation of VraTSR. This is in accordance with previous work showing that reducing the levels of *pbpB* transcription led to higher levels of *vraTSR* transcripts (22). PBP2 is the only PBP whose expression is directly regulated by VraR, a feature shared with MGT (9, 39). This indicates that PBP2 activity is crucial to maintain the VraTSR system in the OFF state. A second common effect of all CW-targeting compounds tested, besides depletion of substrate for PBPs, is the delocalization of PBP2 from the division septum (Fig. S2) (23, 24). Therefore, one possible mechanism mediating the role of PBP2 in VraTSR activation could be a direct interaction between this protein and VraT/VraS, disrupted upon PBP2 delocalization in the presence of CW-targeting compounds. However, this is unlikely to be the case, because we showed that PBP2 delocalization does not correlate with VraTSR activation: the former was observed in the presence of low (1 μg · mL^−1^) concentrations of vancomycin, which were insufficient for VraTSR activation (see Fig. 5). In contrast, inactivation of either the transglycosylase or transpeptidase domain of PBP2 lead to a strong activation of the VraTSR system. Unfortunately, we could not pinpoint which of the two activities had a major role, because inactivation of the transpeptidase domain of PBP2 impaired the activity of the transglycosylase domain, leading to an increased susceptibility to the transglycosylase inhibitor moenomycin. It is possible that processivity of the transglycosylase activity of PBP2 is impaired if the resulting glycans cannot be incorporated into the peptidoglycan mesh via transpeptidation. We favor the hypothesis that it is the decrease of transglycosylase activity that signals cell wall damage because removal/inactivation of the other transpeptidases (PBP1, PBP3, and PBP4) did not result in VraTSR activation; in particular, removal/inactivation of PBP4, whose absence leads to a major decrease in peptidoglycan cross-linking (35). Additionally, lack of MGT, and not of any of the other peptidoglycan synthases tested, also led to VraTSR activation, albeit to a lower level. A decrease in PBP2 transglycosylase activity leads to shorter glycans (40) and therefore to an increase in the number of glycan strand extremities, which could be sensed by VraTSR. Alternatively, a reduction in transglycosylase activity without concomitant reduction of the activity of peptidoglycan hydrolases could lead to an increase in the concentration of a peptidoglycan hydrolysis product that could be sensed as CW damage by VraTSR. Identifying the exact CW damage signal is key to fully understanding the response of MRSA strains to beta-lactam antibiotics.

## MATERIALS AND METHODS

### Bacterial growth conditions.

The plasmids and strains used in this study are listed in Tables S1 and S2 and their construction is described in the supplemental material. S. aureus strains were grown in tryptic soy broth (TSB, Difco) with aeration at 37°C or on tryptic soy agar (TSA, Difco) at 30°C or 37°C. For microscopy experiments, overnight cultures of S. aureus strains were diluted 1:200 in TSB medium and allowed to grow at 37°C until an optical density at 600 nm (OD_600_) of approximately 0.5. Cells were then harvested and resuspended in the same medium. Escherichia coli strains were grown in Luria-Bertani broth (LB, Difco) with aeration, or on LB agar (LA, Difco) at 30°C. The culture medium was supplemented with the appropriate antibiotics (100 μg · mL^−1^ ampicillin, 10 μg · mL^−1^ erythromycin, 10 μg · mL^−1^ chloramphenicol, or 50 μg · mL^−1^ of both kanamycin and neomycin; Sigma-Aldrich), with 100 μg · mL^−1^ 5-bromo-4-chloro-3-indolyl-β-d-galactopyranoside (X-Gal; VWR) or cadmium chloride (0.1 μM, Sigma-Aldrich), when required. Cell wall-targeting antibiotics were used at their MICs: 2 μg · mL^−1^ for vancomycin, 800 μg · mL^−1^ for oxacillin, 1.5 μg · mL^−1^ for CDFI [2-(2-chlorophenyl)-3-[1-(2,3-dimethylbenzyl)piperidin-4-yl]-5-fluoro-1H-indole], 40 μg · mL^−1^ for bacitracin, 125 μg · mL^−1^ for d-cycloserine, 300 μg · mL^−1^ for fosfomycin, and 1 μg · mL^−1^ for PC190273.

### MIC assays.

MICs of relevant antimicrobial compounds were determined by broth microdilution in sterile 96-well plates. Series of 2-fold dilutions of each compound were performed in TSB. Cultures of S. aureus strains and mutants were added at a final density of ~5 × 10^5^ CFU · mL^−1^ to each well. In each plate, some wells were not inoculated, for sterility control, and cell viability was assessed in wells with TSB to which no compound was added. Plates were incubated at 37°C. Endpoints were assessed visually after 24 and 48 h and the MIC was determined as the lowest compound concentration that inhibited growth. All assays were performed in triplicate.

### Fluorescence-activated cell sorting analysis.

Overnight TSB cultures of S. aureus strains were diluted 1:200 in TSB and grown at 37°C to exponential growth phase (OD_600_ of 0.5 to 0.7). The appropriate antibiotic was then added, and cells were incubated for 1 h. After antibiotic incubation, cells were collected by centrifugation, washed once with PBS (phosphate-buffered saline, NaCl 137 mM, KCl 2.7 mM, Na_2_HPO_4_ 10 mM, KH_2_PO_4_ 1.8 mM), and resuspended in PBS. Samples were filtered with a 40-μm pore strainer and diluted 1:50 in PBS. Flow cytometry data were collected on a S3e Cell Sorter (Bio-Rad) using a target flow rate of 500 events per second and collecting 5,000 events for each sample. A 100-mW 488-nm laser line was used for excitation, with amplification settings of 350 forward scattering (FSC) and 950 (FL1, 525/30 nm). Acquisition was triggered by forward scattering with a threshold of 1.15.

### S. aureus imaging by fluorescence microscopy.

For fluorescence microscopy experiments, S. aureus cultures were grown to mid-exponential phase (OD_600_ of 0.5 to 0.7) and 1 mL was collected by centrifugation. Cells were suspended in 30 μL of PBS and 1 μL was placed on a thin layer of agarose (1.2% in PBS). When incubation with antibiotics was required prior to imaging, S. aureus cells were grown to an OD_600_ of 0.3 to 0.4 at which point appropriate compounds were added. Cells were further incubated for 60 min at 37°C, before being collected by centrifugation.

To evaluate septal enrichment when studying protein localization, we determined the fluorescence ratio (FR) calculated as the ratio of the median fluorescence of the 25% brightest pixels of the septum versus median fluorescence at the cell periphery, both corrected for background fluorescence. To measure FRs and cytoplasm fluorescence in two different strains (COL Pvra-sGFP and BCBPM073) incubated for 45 min with increasing concentrations of vancomycin (0, 1, 5 or 10 μg mL^−1^), strains were imaged on the same microscopy slide. For this, COL Pvra-sGFP cells were labeled with DNA dye Hoechst 33342 (1 μg mL^−1^), both cultures were washed with TSB, and then the two cultures were mixed prior to visualization by epifluorescence microscopy.

Microscopy was done using a Zeiss Axio Observer microscope with a Plan-Apochromat 100×/1.4 oil Ph3 objective. Images were acquired with a Retiga R1 CCD camera (QImaging) using Metamorph 7.5 software (Molecular Devices).

### Determination of VraT topology using PhoB fusions.

Overnight cultures of wild-type COL, COLΔ*phoB*, COLΔ*phoB*pCN51, COLΔ*phoB*pVraT-PhoB and COLΔ*phoB*pPhoB-VraT were diluted in TSB to a final OD_600_ of 0.05 and grown to mid-exponential phase (OD_600_ ~ 0.5). Subsequently, 1 mL of culture was harvested by centrifugation and resuspended in the same volume of TSB. Serial 10-fold dilutions of each strain were made and 20 μL of 10^0^, 10^−2^, 10^−4^, and 10^−6^ dilutions were plated onto tryptic soy agar plates containing BCIP (Sigma-Aldrich), supplemented with erythromycin 10 μg · mL^−1^ and 2 μM cadmium chloride (Sigma-Aldrich), when appropriate. Plates were incubated at 37°C for 24h.

## References

[1] Murray CJ, Ikuta KS, Sharara F, Swetschinski L, Robles Aguilar G, Gray A, Han C, Bisignano C, Rao P, Wool E, Johnson SC, Browne AJ, Chipeta MG, Fell F, Hackett S, Haines-Woodhouse G, Kashef Hamadani BH, Kumaran EAP, McManigal B, Agarwal R, Akech S, Albertson S, Amuasi J, Andrews J, Aravkin A, Ashley E, Bailey F, Baker S, Basnyat B, Bekker A, Bender R, Bethou A, Bielicki J, Boonkasidecha S, Bukosia J, Carvalheiro C, Castañeda-Orjuela C, Chansamouth V, Chaurasia S, Chiurchiù S, Chowdhury F, Cook AJ, Cooper B, Cressey TR, Criollo-Mora E, Cunningham M, Darboe S, Day NPJ, De Luca M, Dokova K, et al. 2022. Global burden of bacterial antimicrobial resistance in 2019: a systematic analysis. Lancet 399:629–655. 10.1016/S0140-6736(21)02724-0. 35065702 PMC8841637

[2] Lowy FD. 1998. *Staphylococcus aureus* infections. N Engl J Med 339:520–532. 10.1056/NEJM199808203390806. 9709046

[3] Villanueva M, García B, Valle J, Rapún B, Ruiz de Los Mozos I, Solano C, Martí M, Penadés JR, Toledo-Arana A, Lasa I. 2018. Sensory deprivation in *Staphylococcus aureus*. Nat Commun 9:523. 10.1038/s41467-018-02949-y. 29410457 PMC5802764

[4] Krell T, Lacal J, Busch A, Silva-Jiménez H, Guazzaroni ME, Ramos JL. 2010. Bacterial sensor kinases: diversity in the recognition of environmental signals. Annu Rev Microbiol 64:539–559. 10.1146/annurev.micro.112408.134054. 20825354

[5] Jacob-Dubuisson F, Mechaly A, Betton JM, Antoine R. 2018. Structural insights into the signalling mechanisms of two-component systems. Nat Rev Microbiol 16:585–593. 10.1038/s41579-018-0055-7. 30008469

[6] Zschiedrich CP, Keidel V, Szurmant H. 2016. Molecular mechanisms of two-component signal transduction. J Mol Biol 428:3752–3775. 10.1016/j.jmb.2016.08.003. 27519796 PMC5023499

[7] Boyle-Vavra S, Yin S, Jo DS, Montgomery CP, Daum RS. 2013. VraT/YvqF is required for methicillin resistance and activation of the VraSR regulon in *Staphylococcus aureus*. Antimicrob Agents Chemother 57:83–95. 10.1128/AAC.01651-12. 23070169 PMC3535960

[8] Kuroda M, Kuroda H, Oshima T, Takeuchi F, Mori H, Hiramatsu K. 2003. Two-component system VraSR positively modulates the regulation of cell-wall biosynthesis pathway in *Staphylococcus aureus*. Mol Microbiol 49:807–821. 10.1046/j.1365-2958.2003.03599.x. 12864861

[9] Yin S, Daum RS, Boyle-Vavra S. 2006. VraSR two-component regulatory system and its role in induction of *pbp2* and *vraSR* expression by cell wall antimicrobials in *Staphylococcus aureus*. Antimicrob Agents Chemother 50:336–343. 10.1128/AAC.50.1.336-343.2006. 16377706 PMC1346790

[10] Dengler V, Meier PS, Heusser R, Berger-Bächi B, McCallum N. 2011. Induction kinetics of the *Staphylococcus aureus* cell wall stress stimulon in response to different cell wall active antibiotics. BMC Microbiol 11:16. 10.1186/1471-2180-11-16. 21251258 PMC3032642

[11] Belcheva A, Golemi-Kotra D. 2008. A close-up view of the VraSR two-component system: a mediator of *Staphylococcus aureus* response to cell wall damage. J Biol Chem 283:12354–12364. 10.1074/jbc.M710010200. 18326495

[12] Mascher T. 2006. Intramembrane-sensing histidine kinases: a new family of cell envelope stress sensors in Firmicutes bacteria. FEMS Microbiol Lett 264:133–144. 10.1111/j.1574-6968.2006.00444.x. 17064367

[13] Galbusera E, Renzoni A, Andrey DO, Monod A, Barras C, Tortora P, Polissi A, Kelley WL. 2011. Site-specific mutation of *Staphylococcus aureus* VraS reveals a crucial role for the VraR-VraS sensor in the emergence of glycopeptide resistance. Antimicrob Agents Chemother 55:1008–1020. 10.1128/AAC.00720-10. 21173175 PMC3067069

[14] Belcheva A, Verma V, Korenevsky A, Fridman M, Kumar K, Golemi-Kotra D. 2012. Roles of DNA sequence and sigma A factor in transcription of the VraSR operon. J Bacteriol 194:61–71. 10.1128/JB.06143-11. 22020638 PMC3256607

[15] Utaida S, Dunman PM, Macapagal D, Murphy E, Projan SJ, Singh VK, Jayaswal RK, Wilkinson BJ. 2003. Genome-wide transcriptional profiling of the response of *Staphylococcus aureus* to cell-wall-active antibiotics reveals a cell-wall-stress stimulon. Microbiology (Reading) 149:2719–2732. 10.1099/mic.0.26426-0. 14523105

[16] McCallum N, Meier PS, Heusser R, Berger-BäChi B. 2011. Mutational analyses of open reading frames within the *vraSR* operon and their roles in the cell wall stress response of *Staphylococcus aureus*. Antimicrob Agents Chemother 55:1391–1402. 10.1128/AAC.01213-10. 21220524 PMC3067146

[17] Liu Q, Cho H, Yeo WS, Bae T. 2015. The extracytoplasmic linker peptide of the sensor protein SaeS tunes the kinase activity required for staphylococcal virulence in eesponse to host signals. PLoS Pathog 11:e1004799. 10.1371/journal.ppat.1004799. 25849574 PMC4388633

[18] van Heijenoort J. 1998. Assembly of the monomer unit of bacterial peptidoglycan. Cell Mol Life Sci 54:300–304. 10.1007/s000180050155. 9614964 PMC11147183

[19] Schirner K, Eun Y-J, Dion M, Luo Y, Helmann JD, Garner EC, Walker S. 2015. Lipid-linked cell wall precursors regulate membrane association of bacterial actin MreB. Nat Chem Biol 11:38–45. 10.1038/nchembio.1689. 25402772 PMC4270829

[20] Qiao Y, Srisuknimit V, Rubino F, Schaefer K, Ruiz N, Walker S, Kahne D. 2017. Lipid II overproduction allows direct assay of transpeptidase inhibition by β-lactams. Nat Chem Biol 13:793–798. 10.1038/nchembio.2388. 28553948 PMC5478438

[21] Scheffers D-J, Pinho MG. 2005. Bacterial cell wall synthesis: new insights from localization studies. Microbiol Mol Biol Rev 69:585–607. 10.1128/MMBR.69.4.585-607.2005. 16339737 PMC1306805

[22] Gardete S, Wu SW, Gill S, Tomasz A. 2006. Role of VraSR in antibiotic resistance and antibiotic-induced stress response in *Staphylococcus aureus*. Antimicrob Agents Chemother 50:3424–3434. 10.1128/AAC.00356-06. 17005825 PMC1610096

[23] Pinho MG, Errington J. 2005. Recruitment of penicillin-binding protein PBP2 to the division site of *Staphylococcus aureus* is dependent on its transpeptidation substrates. Mol Microbiol 55:799–807. 10.1111/j.1365-2958.2004.04420.x. 15661005

[24] Mann PA, Müller A, Xiao L, Pereira PM, Yang C, Ho Lee S, Wang H, Trzeciak J, Schneeweis J, Dos Santos MM, Murgolo N, She X, Gill C, Balibar CJ, Labroli M, Su J, Flattery A, Sherborne B, Maier R, Tan CM, Black T, Onder K, Kargman S, Monsma FJ, Pinho MG, Schneider T, Roemer T. 2013. Murgocil is a highly bioactive staphylococcal-specific inhibitor of the peptidoglycan glycosyltransferase enzyme MurG. ACS Chem Biol 8:2442–2451. 10.1021/cb400487f. 23957438

[25] Ostash B, Walker S. 2010. Moenomycin family antibiotics: chemical synthesis, biosynthesis, and biological activity. Nat Prod Rep 27:1594–1617. 10.1039/c001461n. 20730219 PMC2987538

[26] Stock AM, Robinson VL, Goudreau PN. 2000. Two-component signal transduction. Annu Rev Biochem 69:183–215. 10.1146/annurev.biochem.69.1.183. 10966457

[27] Falord M, Karimova G, Hiron A, Msadek T. 2012. GraXSR proteins interact with the VraFG ABC transporter to form a five-component system required for cationic antimicrobial peptide sensing and resistance in *Staphylococcus aureus*. Antimicrob Agents Chemother 56:1047–1058. 10.1128/AAC.05054-11. 22123691 PMC3264281

[28] Hiron A, Falord M, Valle J, Débarbouillé M, Msadek T. 2011. Bacitracin and nisin resistance in *Staphylococcus aureus*: a novel pathway involving the BraS/BraR two-component system (SA2417/SA2418) and both the BraD/BraE and VraD/VraE ABC transporters. Mol Microbiol 81:602–622. 10.1111/j.1365-2958.2011.07735.x. 21696458

[29] Li M, Cha DJ, Lai Y, Villaruz AE, Sturdevant DE, Otto M. 2007. The antimicrobial peptide-sensing system *aps* of *Staphylococcus aureus*. Mol Microbiol 66:1136–1147. 10.1111/j.1365-2958.2007.05986.x. 17961141

[30] Jordan S, Hutchings MI, Mascher T. 2008. Cell envelope stress response in Gram-positive bacteria. FEMS Microbiol Rev 32:107–146. 10.1111/j.1574-6976.2007.00091.x. 18173394

[31] Murakami K, Fujimura T, Doi M. 1994. Nucleotide sequence of the structural gene for the penicillin-binding protein 2 of *Staphylococcus aureus* and the presence of a homologous gene in other staphylococci. FEMS Microbiol Lett 117:131–136. 10.1111/j.1574-6968.1994.tb06754.x. 8181715

[32] Hartman BJ, Tomasz A. 1984. Low-affinity penicillin-binding protein associated with β-lactam resistance in *Staphylococcus aureus*. J Bacteriol 158:513–516. 10.1128/jb.158.2.513-516.1984. 6563036 PMC215458

[33] Pereira SFF, Henriques AO, Pinho MG, De Lencastre H, Tomasz A. 2007. Role of PBP1 in cell division of *Staphylococcus aureus*. J Bacteriol 189:3525–3531. 10.1128/JB.00044-07. 17307860 PMC1855886

[34] Reichmann NT, Tavares AC, Saraiva BM, Jousselin A, Reed P, Pereira AR, Monteiro JM, Sobral RG, VanNieuwenhze MS, Fernandes F, Pinho MG. 2019. SEDS-bPBP pairs direct lateral and septal peptidoglycan synthesis in *Staphylococcus aureus*. Nat Microbiol 4:1368–1377. 10.1038/s41564-019-0437-2. 31086309

[35] Memmi G, Filipe SR, Pinho MG, Fu Z, Cheung A. 2008. *Staphylococcus aureus* PBP4 is essential for β-lactam resistance in community-acquired methicillin-resistant strains. Antimicrob Agents Chemother 52:3955–3966. 10.1128/AAC.00049-08. 18725435 PMC2573147

[36] Pinho MG, De Lencastre H, Tomasz A. 2000. Cloning, characterization, and inactivation of the gene *pbpC*, encoding penicillin-binding protein 3 of *Staphylococcus aureus*. J Bacteriol 182:1074–1079. 10.1128/JB.182.4.1074-1079.2000. 10648534 PMC94384

[37] Wang QM, Peery RB, Johnson RB, Alborn WE, Yeh WK, Skatrud PL. 2001. Identification and characterization of a monofunctional glycosyltransferase from *Staphylococcus aureus*. J Bacteriol 183:4779–4785. 10.1128/JB.183.16.4779-4785.2001. 11466281 PMC99532

[38] Reed P, Veiga H, Jorge AM, Terrak M, Pinho MG. 2011. Monofunctional transglycosylases are not essential for *Staphylococcus aureus* cell wall synthesis. J Bacteriol 193:2549–2556. 10.1128/JB.01474-10. 21441517 PMC3133172

[39] Sengupta M, Jain V, Wilkinson BJ, Jayaswal RK. 2012. Chromatin immunoprecipitation identifies genes under direct VraSR regulation in *Staphylococcus aureus*. Can J Microbiol 58:703–708. 10.1139/w2012-043. 22571705

[40] Pinho MG, De Lencastre H, Tomasz A. 2001. An acquired and a native penicillin-binding protein cooperate in building the cell wall of drug-resistant staphylococci. Proc Natl Acad Sci USA 98:10886–10891. 10.1073/pnas.191260798. 11517340 PMC58569

[41] Manoil C, Beckwith J. 1986. A genetic approach to analyzing membrane protein topology. Science 233:1403–1408. 10.1126/science.3529391. 3529391

[42] Kahan FM, Kahan JS, Cassidy PJ, Kropp H. 1974. The mechanism of action of fosfomycin (phosphonomycin). Ann N Y Acad Sci 235:364–386. 10.1111/j.1749-6632.1974.tb43277.x. 4605290

[43] Lambert MP, Neuhaus FC. 1972. Mechanism of d-cycloserine action: alanine racemase from *Escherichia coli* W. J Bacteriol 110:978–987. 10.1128/jb.110.3.978-987.1972. 4555420 PMC247518

[44] Huber J, Donald RGK, Lee SH, Jarantow LW, Salvatore MJ, Meng X, Painter R, Onishi RH, Occi J, Dorso K, Young K, Park YW, Skwish S, Szymonifka MJ, Waddell TS, Miesel L, Phillips JW, Roemer T. 2009. Chemical genetic identification of peptidoglycan inhibitors potentiating carbapenem activity against methicillin-resistant *Staphylococcus aureus*. Chem Biol 16:837–848. 10.1016/j.chembiol.2009.05.012. 19716474

[45] Stone KJ, Strominger JL. 1971. Mechanism of action of bacitracin: complexation with metal ion and C(55)-isoprenyl pyrophosphate. Proc Natl Acad Sci USA 68:3223–3227. 10.1073/pnas.68.12.3223. 4332017 PMC389626

[46] Beauregard DA, Williams DH, Gwynn MN, Knowles DJC. 1995. Dimerization and membrane anchors in extracellular targeting of vancomycin group antibiotics. Antimicrob Agents Chemother 39:781–785. 10.1128/AAC.39.3.781. 7793894 PMC162627

[47] Ghuysen JM. 1991. Serine β-lactamases and penicillin-binding proteins. Annu Rev Microbiol 45:37–67. 10.1146/annurev.mi.45.100191.000345. 1741619

