# Supplementary material for: Revisiting the Role of VraTSR in Staphylococcus aureus Response to Cell Wall-Targeting Antibiotics
Source: J Bacteriol. 2022 Jul 13;204(8):e00162-22. doi: 10.1128/jb.00162-22 (PMC9380581; doi:10.1128/jb.00162-22)
Supplement: Supplemental file 1 — Supplemental material. Download jb.00162-22-s0001.pdf, PDF file, 0.5 MB [file jb.00162-22-s0001.pdf]

Revisiting the role of VraTSR in *Staphylococcus aureus* response to cell wall targeting antibiotics

Supplemental Information

Pedro B. Fernandes, Patricia Reed, João M. Monteiro and Mariana G. Pinho,

Supplemental Methods

Construction of *S. aureus* strains

Sequence of primers used for strain construction as listed in Supplemental Table 3.

To construct the *vraTSR* promoter fusion, an 814-bp DNA fragment containing the *vraTSR* promoter region was amplified from COL genomic DNA using primers PvraSR_P1_KpnI and PvraSR_P2_New_XhoI, digested with KpnI and XhoI, and cloned into KpnI/XhoI digested pFAST3, upstream of sfgfp-p7, resulting in plasmid pPvra-pFAST3, confirmed by DNA sequencing. pPvrap-FAST3 was electroporated into the *S. aureus* RN4220 strain and integrated into the chromosome at the *vraTSR* promoter site by homologous recombination, as confirmed by PCR and sequencing; the resulting strain was named RN Pvra-sGFP. Strain COL Pvra-sGFP was constructed by transducing the integrated plasmid pPvra-pFAST3 from RN Pvra-sGFP into COL using phage80α, as previously described[1].

To construct the *S. aureus* Δ*pbpB* null mutant, lacking PBP2, we amplified 1Kb DNA fragments from *S. aureus* COL genomic DNA corresponding to the upstream (primers PBP2_KO-P1 and PBP2_KO-P2) and downstream (primers PBP2_KO-P13 and PBP2_KO-P4) regions of the *pbpB* gene. The resulting PCR products were joined by overlap PCR using primers PBP2_KO-P1 and PBP2_KO-P4. The overlap PCR product was digested with EcoRI and BamHI and cloned into the thermosensitive plasmid pMAD[2], producing plasmid pΔ*pbpB*. The plasmid was sequenced and introduced into RN4220 by electroporation[3]. Following electroporation, the plasmid was transduced into COL using phage 80α

as previously described[1]. Insertion and excision of pΔ*pbpB* was performed as previously described[2], except the integration steps were performed at 37 °C instead of 43 °C, resulting in strain COLΔ*pbpB.* Deletion of the target gene was verified by PCR and resulting strains were verified by whole genome sequencing.

To monitor *vraTSR* expression levels in the mutants lacking different enzymes involved in the last stages of peptidoglycan synthesis, pPvra-pFAST3 was transduced into the corresponding mutant strains, using phage 80α, with erythromycin selection, resulting in strains: ColPBP1TP_Pvra-sGFP, COLΔ*pbpB*_Pvra-sGFP, COLΔ*pbp3*_Pvra-sGFP, COLΔ*pbpD*_Pvra-sGFP, COLΔ*mgt*_Pvra-sGFP, COLΔ*sgtA*_Pvra-sGFP and COLΔ*mecA*_Pvra-sGFP.

To investigate the mechanism behind PBP2-dependent VraTSR activation, COLΔ*pbpB*_Pvra-sGFP was complemented with plasmids encoding different alleles of PBP2. For that, a 2214-bp fragment encompassing the wild type *pbpB* allele was amplified using primers PBP2muts_P1 and PBP2muts_P2 from *S. aureus* COL genomic DNA. The same pair of primers was used to amplify a fragment, coding for the transglycosylase-inactive PBP2^{E114Q} from COLTG42[4] genomic DNA. These fragments were introduced into SmaI-digested pCNX using a Gibson assembly cloning kit (NEB), originating plasmids pPBP2 and pPBP2TG. Transduction of these plasmid into COLΔ*pbpB*_Pvra-sGFP gave COLΔ*pbpB*_Pvra-sGFPpPBP2 and COLΔ*pbpB*_Pvra-sGFPpPBP2TG*, respectively. To construct plasmid pCNX-pbp2TP, a full copy of *pbpB* allele encoding for PBP2^{S398G} (pbp2TP) was cloned into the pCNX plasmid, downstream of the cadmium inducible P_{cad} promoter. The pbp2TP sequence was amplified from pMAD-pbp2TPbig which was constructed by amplifying two PCR fragments encompassing 1.7 kb upstream or downstream of nucleotide T1192 of *pbpB* using primers P1pMADpbp2TP/ P2pMADpbp2TP and P3pMADpbp2TP/ P4pMADpbp2TP, respectively. The two fragments were joined by overlap PCR using primers pair P1pMADpbp2TP and P4pMADpbp2TP, digested with BglII and SmaI and cloned into pMAD, creating plasmid pMADpbp2TPbig. This plasmid contains the nucleotide exchanges T1192G and C1193G which switch the catalytic serine from the TP domain of PBP2 to a glycine (S398G) and a silent mutation (T1197C) that introduces a BamHI restriction site to facilitate screening

of clones. The pMAD-PBP2TPbig plasmid served as a template to amplify the full pbp2TP allele using primers P1pCNXpbp2 and P2pCNXpbp2. The resulting PCR fragment was cloned downstream of the P_{cad} promoter of pCNX plasmid, after digesting with SalI and KpnI, resulting in plasmid pCNX-pbp2TP. pCNX-pbp2TP was then transduced to COLΔ*pbpB*_Pvra-sGFP, originating strain COLΔ*pbpB*_Pvra-sGFPpPBP2TP*. To construct pPBP2TGTP, the mutation coding for transglycosylase-inactive $PBP2^{E114Q}$ was inserted into plasmid pCNX-pbp2TP, via site-directed mutagenesis, using primers E114Q fw and E114Q rev. The resulting plasmid was transduced to COLΔ*pbpB*_Pvra-sGFP, giving strain COL*pbpB*_Pvra-sGFPpPBP2TG*TP*.

To localize VraT and VraS, an N-terminal fusion of VraT to the P7 variant of superfast GFP (sfGFP)[5] and a C-terminal fusion of VraS to mCherry were constructed. A 409-bp fragment encoding the upstream region of *vraT* was amplified by PCR from *S. aureus* COL genomic DNA, using primers orf1_fw_EcoRI and orf1_rev_link_sgfp. Primers sgfp_fw_link_orf1 and sgfp_rev_link_vraT were used to amplify *gfp* from pFAST3. A third 744-bp fragment containing *vraT* and a sequence encoding an 11 amino acid linker was amplified using primers vraT fw_link_sgfp and vraT rev BamHI. The fragments were joined by overlap PCR using the primers orf1_fw_EcoRI and vraT rev BamHI. The resulting fragment was digested with EcoRI and BamHI restriction enzymes and cloned into pMAD, giving pVraT-GFP. The plasmid was sequenced and then electroporated into *S. aureus* RN4220 strain at 30 °C, using erythromycin and X-gal selection, and transduced to COL using phage 80α. Integration and excision of the plasmid into the chromosome was performed as previously described[2], giving COLsGFP_VraT. An 862-bp fragment encoding VraS, excluding the stop codon, and a sequence encoding an 11 amino acid linker was amplified by PCR from *S. aureus* COL genomic DNA, using primers VraSCterfwdNcoI and VraSmchMLbwd. Primers mCherryMLfwd and mCherrybwd2 were used to amplify *mCherry* from pBCB4-Cherry. A third 860-bp fragment containing the downstream region of *vraS* was amplified using primers Vrasfwd3 and VraRbwdBamHI. The fragments were joined by overlap PCR using the primers mCherryMLfwd and VraRbwdBamHI. The resulting fragment was digested with NcoI and BamHI restriction enzymes and cloned into pMAD, giving

pVraS-mCherry. The plasmid was sequenced and then electroporated into *S. aureus* RN4220 strain at 30 °C, using erythromycin selection in the presence of X-gal, and transduced into COLsGFP_VraT, using phage 80α. Integration and excision of the plasmid into the chromosome was performed as described[2], resulting in strain COLsGFP-VraTVraS-mCherry.

To determine the topology of VraT, PhoB fusions to VraT were made at both N- and C-terminal ends. For the N-terminal fusion to VraT a 1389-bp fragment encompassing *phoB* allele and a 5 amino acid linker was amplified using primers phoB_vraT_P1 and phoB_vraT_P2 from *S. aureus* COL genomic DNA. Another fragment with 748-bp with *vraT* coding sequence was amplified from the same genomic DNA, using primers phoB_vraT_P3 and phoB_vraT_P4. These fragments were introduced into SmaI-digested pCN51 using a Gibson assembly cloning kit (NEB), originating plasmid pPhoB_VraT. For the C-terminal PhoB fusion to VraT, a 772-bp fragment with *vraT* coding sequence and encoding a 5 amino acid linker was amplified from *S. aureus* COL genomic DNA, using primers vraT_phoB_P1 and vraT_phoB_P2. Primers vraT_phoB_P3 and vraT_phoB_P4 were used to amplify a 1376-bp DNA fragment with *phoB* coding sequence, from the same genomic template. These fragments were introduced into SmaI-digested pCN51 using a Gibson assembly cloning kit (NEB), originating plasmid pVraT-PhoB. Plasmids pCN51, pPhoB_VraT and pVraT-PhoB were transduced to COLΔ*phoB*, giving strains COLΔ*phoB*pCN51, COLΔ*phoB*pPhoB-VraT and COLΔ*phoB*pVraT-PhoB, respectively.

Supplemental Figures

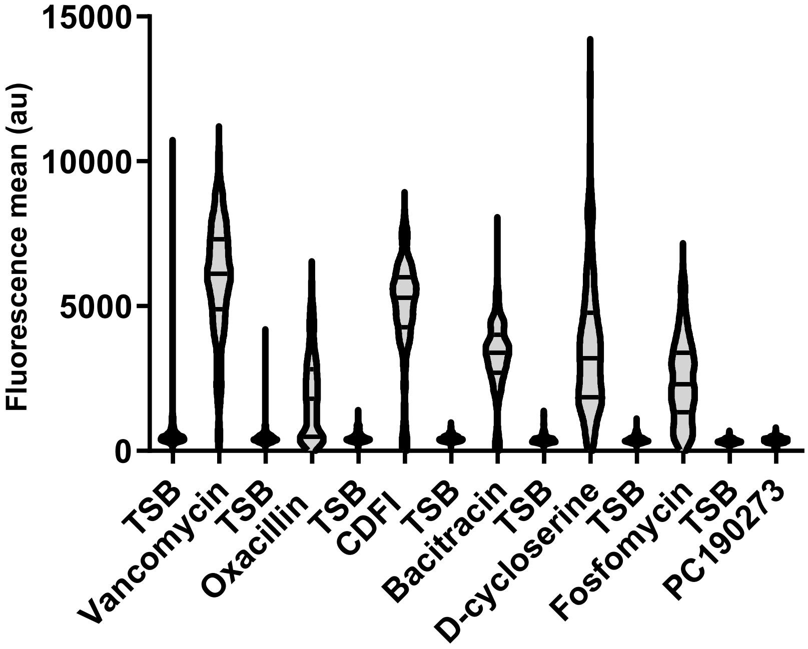

Supplementary Figure 1. Blocking early, middle or late stages of CW synthesis results in VraTSR activation. *S. aureus* cells expressing GFP under the control of the *vraTSR* promoter (COL Pvra-sGFP) were incubated with 1x MIC of different CW targeting antibiotics for 60 mins prior to imaging by fluorescence microscopy. Cells from a control experiment, with no antibiotic, were always present on the same slide and those cells were labelled with DNA dye Hoechst 33342, to discriminate the two populations. Incubation with the FtsZ inhibitor PC190273, an antibiotic that does not target the CW synthesis, was also included as a control. N > 223 cells for each condition. Data represented in violin plots where the middle line represents the median and the other two lines the quartiles.

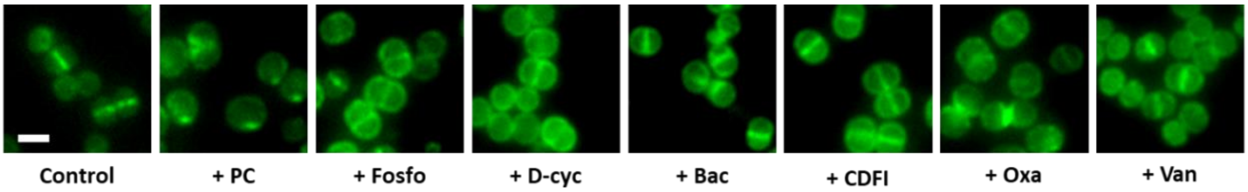

Supplementary Figure 2. PBP2 delocalizes in the presence of cell wall targeting antibiotics. COL *pbpB*::*sgfp-pbpB* (BCBPM073) cells were imaged by epifluorescence microscopy after incubation for 60 min with 1 x MIC of PC190273, (+PC, 1 µg mL^{-1}), fosfomycin (+Fosfo, 300 µg mL^{-1}), D-cycloserine (+D-cyc, 125 µg mL^{-1}), bacitracin (+Bac, 40 µg mL^{-1}), 2-(2-Chlorophenyl)-3-[1-(2,3-dimethylbenzyl)piperidin-4-yl]-5-fluoro-1H-indole (+CDFI,1.5 µg mL^{-1}), oxacillin (+Oxa, 800 µg mL^{-1}) and vancomycin (+Van, 2 µg mL^{-1}). Incubation in TSB without antibiotics was used as negative control. In the presence of all antibiotics tested, septal enrichment of PBP2 is lost or decreased and the protein becomes dispersed over the membrane. Scale bar = 1µm

Supplemental Tables

Supplementary Table 1. Plasmids used in this study

Plasmids	Description	Source or reference
pCN51	Shuttle vector containing a cadmium inducible Pcad promoter; Amp^R Ery^R	6
pCNX	Shuttle vector containing a cadmium inducible Pcad promoter; Amp^R Kan^R	7
pMAD	*E. coli-S. aureus* shuttle vector with a thermosensitive origin of replication for Gram-positive bacteria; Amp^R Ery^R *lacZ*	2
pBCB4-ChE	*S. aureus* integrative vector for N- and C-terminal mCherry fusions; Amp^R Ery^R	8
pFAST3	*S. aureus* integrative vector that allows for C-terminal sGFP fusions; Amp^R Ery^R	9
pPvra-pFAST3	*S. aureus* integrative vector with *vraTSR* promoter upstream of sfgfp-p7	This study
pPBP2	pCNX derivative containing *pbpB* under the control of Pcad promoter; Amp^R Kan^R	This study
pPBP2TG	pCNX derivative containing *pbpB*TG (E114Q) under the control of Pcad promoter; Amp^R Kan^R	This study
pCNX-pbp2TP	pCNX derivative containing *pbp2*TP (S398G) under the control of Pcad promoter; Amp^R Kan^R	This study
pPBP2TGTP	pCNX derivative containing pbp2TGTP (E114Q and S398G) under the control of Pcad promoter; Amp^R Kan^R	This study
pVraT-GFP	pMAD with s*gfp* 5´ fusion to *vraT*, Amp^R, Ery^R	This study
pVraS-mCherry	pMAD with *mCherry* 3' fusion to *vraS,* Amp^R, Ery^R	This study
pVraT-PhoB	pCN51 derivative containing *phoB* 3´ fusion to *vraT* under the control of Pcad promoter, Amp^R, Ery^R	This study
pPhoB_VraT	pCN51 derivative containing *phoB* 5' fusion to *vraT* under the control of Pcad promoter, Amp^R, Ery^R	This study

Supplementary Table 2. Strains used in this study.

Strains	Description	Source or reference
Escherichia coli		
DC10B	Δ*dcm* in the DH10B background; Dam methylation only	10
Staphylococcus aureus		
COL	HA-MRSA	11
RN4220	Restriction-deficient derivative of NCTC8325-4	12
COL Pvra-sGFP	COL with pFAST3-Pvra; Ery^R	This study
ColPBP1TP	COL tet^S *pbpA*::$pbpA^{S314A}$	13
COLΔ*pbpB*	*pbpB* deletion in COL	This study
COLΔ*pbp3*	*pbp3* deletion in COL	13
COLΔ*pbpD*	*pbpD* deletion in COL	14
COLΔ*mgt*	*mgt* deletion in COL	15
COLlΔ*sgtA*	*sgtA* deletion in COL	15
COLΔ*mecA*	*mecA* deletion in COL	16
ColPBP1TP_Pvra-sGFP	COL tet^S *pbpA::*$pbpA^{S314A}$ with pPvra-pFAST3; Ery^R	This study
COLΔ*pbpB*_Pvra-sGFP	COLΔ*pbpB* with pPvra-pFAST3; Ery^R	This study
COLΔ*pbp3*_Pvra-sGFP	COLΔ*pbpC* with pPvra-pFAST3; Ery^R	This study
COLΔ*pbpD*_Pvra-sGFP	COLΔ*pbpD* with pPvra-pFAST3; Ery^R	This study
COLΔ*mgt*_Pvra-sGFP	COLΔ*mgt* with pPvra-pFAST3; Ery^R	This study
COLΔ*sgtA*_Pvra-sGFP	COLΔ*sgtA* with pPvra-pFAST3; Ery^R	This study
COLΔ*mecA*_Pvra-sGFP	COLΔ*mecA* with pPvra-pFAST3; Ery^R	This study
COLΔ*pbpB*_Pvra-sGFPpCNX	COLΔ*pbpB*_Pvra-sGFP with pCNX; Ery^R, Kan^R	This study
COLΔ*pbpB*_Pvra-sGFPpPBP2	COLΔ*pbpB*_Pvra-sGFP with pPBP2; Ery^R, Kan^R	This study
COLΔ*pbpB*_Pvra-sGFPpPBP2TG*	COLΔ*pbpB*_Pvra-sGFP with pPBP2TG; Ery^R, Kan^R	This study
COLΔ*pbpB*_Pvra-sGFPpPBP2TP*	COLΔ*pbpB*_Pvra-sGFP with pCNX-pbp2TP; Ery^R, Kan^R	This study
COLΔ*pbpB*_Pvra-sGFPpPBP2TG*TP*	COLΔ*pbpB*_Pvra-sGFP and pPBP2TGTP, Ery^R, Kan^R	This study
BCBPM073	COL *pbpB*::*sgfp-pbpB*	17
COLsGFP_VraT	COL *vraT*::*sgfp-vraT*	This study
COLsGFP-VraTVraS-mCherry	COL *vraT*::*sgfp-vraT, vraS::vraS-mcherry*	This study
COLΔ*phoB*	*phoB* deletion in COL	Veiga & Pinho

COLΔ*phoB*pCN51	ColΔ*phoB* with pCN51; Ery^R	This study
COLΔ*phoB*pVraT-PhoB	ColΔ*phoB* with pVraT_PhoB; Ery^R	This study
COLΔ*phoB*pPhoB-VraT	ColΔ*phoB* with pPhoB_VraT; Ery^R	This study

Supplementary Table 3. Oligonucleotides used in this study

Primer name	Sequence (5'-3')
PVraSR_P1_KpnI	gctgcggtacccggtgctatttctgcgcc
PVraSR_P2_New_Xho	cgcgctcgagttataataagttttaaaataccaaatgcgc
PBP2KO-P1	acgacgaattctcaaatacacttctgctg
PBP2KO-P2	tagttgaatatatccgtcatacgcggtcctcactttc
PBP2KO-P3	tgaggaccgcgtatgacggatatattcaactaatc
PBP2KO-P4	acgcaggattctgtccactattagaagattg
PBP2muts_P1	ggtcgactctagaggatccccgttgatattagatga
	aagtgaggaccgcgtatgacggaaaacaaaggatc
PBP2muts_P2	gcctgaattcgagctcggtacccttagttgaatatacctgttaatcc
P1pMADpbp2TP	gcgcccgggcattcaaatacgttttatc
P2pMADpbp2TP	cgcggatccaccagtagggtgaggatc
P3pMADpbp2TP	cgcggatccttaaaacctttcttagcg
P4pMADpbp2TP	cgcgtcgacgaattttaaagaaagatatg
P1pCNXpbp2	caggtcgacaaaaaataaggaggaaaaaaaatgacggaaaacaaaggatcttctc
P2pCNXpbp2	cgcatggtaccttagttgaatatacctgttaatccaccgctgtttc
E114Q fw	gtactcgcgactcaagacaatcgtttctacgaacatg
E114Q rev	cgattgtcttgagtcgcgagtactgcgtctttc
VraSCterfwdNcoI	cgcgcgccatggggtactcgcttacaaaatcaatcagc
VraSmchMLbwd	agaaccagcagcggagccagccgaatttaaaggtgctttcacc
mCherryMLfwd	tccgctgctggttctggcgagttcatgattgtgagcaagg
mCherrybwd2	atacgaatcctccttctagtacagctcgtccatgccaccgg
Vrasfwd3	aaggaggattcgtatgacgattaaagtattgtttgtggatg
VraRbwdBamHI	gcgcgcggatcctatcatgttgatgaacatcctagtgatacc
orf1_fw_EcoRI	gcgcgaattcaaggtgatagttatgaactatgttgaacg
orf1_rev_link_sgfp	ctatgccacagcgttcatataatttattagatacg
sgfp_fw_link_orf1	gaacgctgtggcataggatatcataaggaggattcg
sgfp_rev_link_vraT	gaactcgccagaaccagcagcggagccagccgaatgatggtgatggtcgactttg
vraT fw_link_sgfp	tcggctggctccgctgctggttctggcgagttcatgacacacaaatatatatc
vraT rev bamHI	gcgcggatcctcatcgataaatcacctctacgtctccgataaacg
phoB_vraT_P1	gtcgactctagaggatccccctcaggaggatgtcttatgcaatccgataaaagttctaaagatg
phoB_vraT_P2	catggaggcgccgcaggacttgaatatatcaaatattatttttgcgc
phoB_vraT_P3	caagtcctgcggcgcctccatgacacacaaatatatatcaacg
phoB_vraT_P4	cctgaattcgagctcggtaccctcaactcatcgataaatcacctctacgtc
vraT_phoB_P1	ggtcgactctagaggatccccgaacgctgtggcatagaaaggcggcgaaac
vraT_phoB_P2	cggattgggaggcgccgcaggatcgataaatcacctctacgtctcc
vraT_phoB_P3	cgatcctgcggcgcctcccaatccgataaaagttctaaag
vraT_phoB_P4	gcctgaattcgagctcggtacccttacttgaatatatcaaatattatttttgcgc

References

1. Oshida T, Tomasz A. Isolation and characterization of a Tn551-autolysis mutant of *Staphylococcus aureus*. *J Bacteriol*. 1992;174(15):4952-4959 doi:10.1128/JB.174.15.4952-4959.1992

2. Arnaud M, Chastanet A, Débarbouillé M. New vector for efficient allelic replacement in naturally nontransformable, low-GC-content, gram-positive bacteria. *Appl Environ Microbiol*. 2004;70(11):6887-6891 doi:10.1128/AEM.70.11.6887-6891.2004

3. Kraemer GR, Iandolo JJ. High-frequency transformation of *Staphylococcus aureus* by electroporation. *Curr Microbiol*. 1990;21(6):373-376 doi:10.1007/BF02199440

4. Pinho MG, de Lencastre H, Tomasz A. An acquired and a native penicillin-binding protein cooperate in building the cell wall of drug-resistant staphylococci. *Proc Natl Acad Sci U S A*. 2001; 98(19):10886-91 doi:10.1073/pnas.191260798

5. Fisher AC, DeLisa MP. Laboratory evolution of fast-folding green fluorescent protein using secretory pathway quality control. *PLoS One*. 2008;3(6):e2351 doi:10.1371/journal.pone.0002351

6. Charpentier E, Anton AI, Barry P, Alfonso B, Fang Y, Novick RP. Novel cassette-based shuttle vector system for gram-positive bacteria. *Appl Environ Microbiol*. 2004;70(10):6076-6085 doi:10.1128/AEM.70.10.6076-6085.2004

7. Monteiro JM, Fernandes PB, Vaz F, *et al*. Cell shape dynamics during the staphylococcal cell cycle. *Nat Commun*. 2015; 6, 8055 doi:10.1038/ncomms9055

8. Pereira PM, Veiga H, Jorge AM, Pinho MG. Fluorescent reporters for studies of cellular localization of proteins in *Staphylococcus aureus*. *Appl Environ Microbiol*. 2010;76(13):4346-4353 doi:10.1128/AEM.00359-10

9. Nair DR, Monteiro JM, Memmi G, *et al*. Characterization of a novel small molecule that potentiates β-lactam activity against gram-positive and gram-negative pathogens. *Antimicrob Agents Chemother*. 2015;59(4):1876-1885 doi:10.1128/AAC.04164-14

10. Monk IR, Shah IM, Xu M, Tan MW, Foster TJ. Transforming the untransformable: Application of direct transformation to manipulate genetically *Staphylococcus aureus* and *Staphylococcus epidermidis*. *MBio*. 2012;3(2) doi:10.1128/mBio.00277-11

11. Gill SR, Fouts DE, Archer GL, *et al.* Insights on evolution of virulence and resistance from the complete genome analysis of an early methicillin-resistant *Staphylococcus aureus* strain and a biofilm-producing methicillin-resistant *Staphylococcus epidermidis* strain. *J Bacteriol*. 2005;187(7):2426-2438 doi:10.1128/JB.187.7.2426-2438.2005

12. Nair D, Memmi G, Hernandez D, *et al*. Whole-genome sequencing of

Staphylococcus aureus strain RN4220, a key laboratory strain used in virulence research, identifies mutations that affect not only virulence factors but also the fitness of the strain. *J Bacteriol*. 2011;193(9):2332-2335. doi:10.1128/JB.00027-11

13. Reichmann NT, Tavares AC, Saraiva BM, *et al*. SEDS–bPBP pairs direct lateral and septal peptidoglycan synthesis in *Staphylococcus aureus*. *Nat Microbiol*. 2019;4(8):1368-1377 doi:10.1038/s41564-019-0437-2

14. Memmi G, Filipe SR, Pinho MG, Fu Z, Cheung A. *Staphylococcus aureus* PBP4 is essential for β-lactam resistance in community-acquired methicillin-resistant strains. *Antimicrob Agents Chemother*. 2008;52(11):3955-3966. doi:10.1128/AAC.00049-08

15. Reed P, Veiga H, Jorge AM, Terrak M, Pinho MG. Monofunctional transglycosylases are not essential for *Staphylococcus aureus* cell wall synthesis. *J Bacteriol*. 2011;193(10):2549-2556. doi:10.1128/JB.01474-10

16. Reed P, Atilano ML, Alves R, *et al*. *Staphylococcus aureus* survives with a minimal peptidoglycan synthesis machine but sacrifices virulence and antibiotic resistance. *PLoS Pathog*. 2015;11(5):e1004891 doi:10.1371/journal.ppat.1004891

17. Tan CM, Therien AG, Lu J, *et al*. Restoring methicillin-resistant *Staphylococcus aureus* susceptibility to β-lactam antibiotics. *Sci Transl Med*. 2012;4(126) doi:10.1126/scitranslmed.3003592
